# 1-[1-(4-Chloro­phen­yl)ethyl­idene]carbono­hydrazide

**DOI:** 10.1107/S1600536809030384

**Published:** 2009-08-12

**Authors:** Lingyun Du, Lei Du, Shuhao Wang

**Affiliations:** a College of Chemistry and Chemical Engineering, Liaocheng University, Shandong 252059, People’s Republic of China

## Abstract

The mol­ecular skeleton of the title mol­ecule, C_9_H_11_ClN_4_O, is essentially planar, the dihedral angle between the ring and the and N/N/C plane being 6.7 (3)°. In the crystal, inter­molecular N—H⋯O and N—H⋯N hydrogen bonds link the mol­ecules into ribbons propagated along [010].

## Related literature

For the biological activity of carbonohydrazide derivatives, see: Loncle *et al.* (2004 ▶). For related structures, see Meyers *et al.* (1995 ▶).
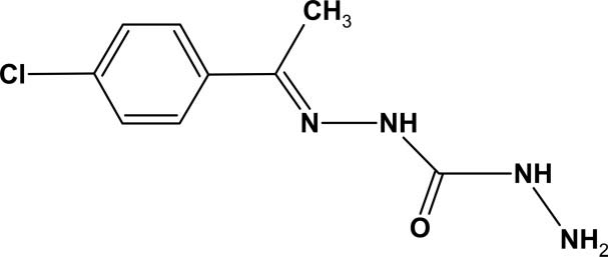


## Experimental

### 

#### Crystal data

C_9_H_11_ClN_4_O
*M* *_r_* = 226.67
Monoclinic, 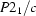

*a* = 14.6429 (14) Å
*b* = 9.6041 (12) Å
*c* = 7.4327 (9) Å
β = 90.102 (1)°
*V* = 1045.3 (2) Å^3^
*Z* = 4
Mo *K*α radiation
μ = 0.34 mm^−1^
*T* = 298 K
0.40 × 0.30 × 0.12 mm

#### Data collection

Bruker SMART APEX CCD area-detector diffractometer
Absorption correction: multi-scan (*SADABS*; Sheldrick, 1996 ▶) *T*_min_ = 0.875, *T*_max_ = 0.960
4419 measured reflections
1837 independent reflections
1085 reflections with *I* > 2σ(*I*)
*R*_int_ = 0.037

#### Refinement

*R*[*F* ^2^ > 2σ(*F* ^2^)] = 0.045
*wR*(*F* ^2^) = 0.126
*S* = 1.01
1837 reflections
137 parameters
H-atom parameters constrained
Δρ_max_ = 0.21 e Å^−3^
Δρ_min_ = −0.20 e Å^−3^

### 

Data collection: *SMART* (Siemens, 1996 ▶); cell refinement: *SAINT* (Siemens, 1996 ▶); data reduction: *SAINT*; program(s) used to solve structure: *SHELXS97* (Sheldrick, 2008 ▶); program(s) used to refine structure: *SHELXL97* (Sheldrick, 2008 ▶); molecular graphics: *SHELXTL* (Sheldrick, 2008 ▶); software used to prepare material for publication: *SHELXTL*.

## Supplementary Material

Crystal structure: contains datablocks I, global. DOI: 10.1107/S1600536809030384/cv2587sup1.cif

Structure factors: contains datablocks I. DOI: 10.1107/S1600536809030384/cv2587Isup2.hkl

Additional supplementary materials:  crystallographic information; 3D view; checkCIF report

## Figures and Tables

**Table 1:** Hydrogen-bond geometry (Å, °)

*D*—H⋯*A*	*D*—H	H⋯*A*	*D*⋯*A*	*D*—H⋯*A*
N1—H1⋯N4^i^	0.86	2.24	3.024 (3)	152
N3—H3⋯O1^ii^	0.86	2.09	2.850 (3)	147
N4—H4*A*⋯O1^iii^	0.89	2.34	3.206 (3)	164

Symmetry codes: (i) 
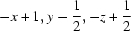
; (ii) 
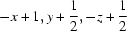
; (iii) 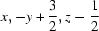.

**Fig. 1.**
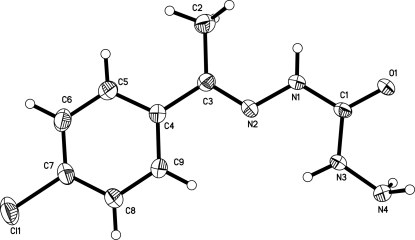
The molecular structure of (I) showing the atomic numbering scheme and 30% probability displacement ellipsoids.

## supplementary crystallographic information

### Comment

A number of carbonohydrazide derivatives have been claimed to possess a bioactivity such as antibacterial, antifungal, anticonvulsant and anticancer activities (Loncle *et al.*, 2004). We describe in this paper a user-friendly, solvent-free protocol for the synthesis of substituted carbonohydrazide starting from the fragrant ketones and carbohydrazide under solvent-free conditions in this paper. Using this method, which can be considered as a a general method for the synthesis of substituted carbonohydrazides, we obtained the title compound, (I). We present here its crystal structure.

In (I) (Fig. 1), the bond lengths and angles are normal and correspond to those observed in bis(3-fluorophenylmethine)carbonohydrazide (Meyers *et al.*, 1995). The N4/N3/C1 and N2/N1/C1 planes form a dihedral angle of 4.09 (4)°, while ring C4-C9 and N2/N1/C1 plane form a dihedral angle of 2.64 (29)°.

In the crystal, intermolecular N—H···O and N—H···N hydrogen bonds (Table 1) link the molecules into ribbons propagated in direction [010].

### Experimental

*p*-Chloroacetophenone (5.0 mmol) and carbohydrazide (5.0 mmol) were mixed in 50 ml flash under sovlent-free condtions After stirring 3 h at 373 K, the resulting mixture was cooled to room temperature, and recrystalized from ethanol, and afforded the title compound as a crystalline solid. Elemental analysis: calculated for C_9_H_11_ClN_4_O: C 47.69, H 4.89, N 24.72%; found: C 47.63, H 4.75, N 24.64%.

### Refinement

All H atoms were placed in geometrically idealized positions (N—H 0.86 and C—H = 0.93–0.96 Å) and treated as riding on their parent atoms, with *U*_iso_(H) = 1.2–1.5 *U*_eq_(C) (C,*N*).

### Crystal data

**Table:**

C_9_H_11_ClN_4_O	*F*(000) = 472
*M**_r_* = 226.67	*D*_x_ = 1.440 Mg m^−^^3^
Monoclinic, *P*2_1_/*c*	Mo *K*α radiation, λ = 0.71073 Å
*a* = 14.6429 (14) Å	Cell parameters from 963 reflections
*b* = 9.6041 (12) Å	θ = 2.5–22.7°
*c* = 7.4327 (9) Å	µ = 0.34 mm^−^^1^
β = 90.102 (1)°	*T* = 298 K
*V* = 1045.3 (2) Å^3^	Needle, colourless
*Z* = 4	0.40 × 0.30 × 0.12 mm

### Data collection

**Table:**

Bruker SMART APEX CCD area-detector diffractometer	1837 independent reflections
Radiation source: fine-focus sealed tube	1085 reflections with *I* > 2σ(*I*)
graphite	*R*_int_ = 0.037
φ and ω scans	θ_max_ = 25.0°, θ_min_ = 1.4°
Absorption correction: multi-scan (*SADABS*; Sheldrick, 1996)	*h* = −11→17
*T*_min_ = 0.875, *T*_max_ = 0.960	*k* = −11→11
4419 measured reflections	*l* = −7→8

### Refinement

**Table:**

Refinement on *F*^2^	Primary atom site location: structure-invariant direct methods
Least-squares matrix: full	Secondary atom site location: difference Fourier map
*R*[*F*^2^ > 2σ(*F*^2^)] = 0.045	Hydrogen site location: inferred from neighbouring sites
*wR*(*F*^2^) = 0.126	H-atom parameters constrained
*S* = 1.01	*w* = 1/[σ^2^(*F*_o_^2^) + (0.0508*P*)^2^ + 0.3562*P*] where *P* = (*F*_o_^2^ + 2*F*_c_^2^)/3
1837 reflections	(Δ/σ)_max_ = 0.002
137 parameters	Δρ_max_ = 0.21 e Å^−^^3^
0 restraints	Δρ_min_ = −0.20 e Å^−^^3^

### Special details

**Table:**

Geometry. All e.s.d.'s (except the e.s.d. in the dihedral angle between two l.s. planes) are estimated using the full covariance matrix. The cell e.s.d.'s are taken into account individually in the estimation of e.s.d.'s in distances, angles and torsion angles; correlations between e.s.d.'s in cell parameters are only used when they are defined by crystal symmetry. An approximate (isotropic) treatment of cell e.s.d.'s is used for estimating e.s.d.'s involving l.s. planes.
Refinement. Refinement of *F*^2^ against ALL reflections. The weighted *R*-factor *wR* and goodness of fit *S* are based on *F*^2^, conventional *R*-factors *R* are based on *F*, with *F* set to zero for negative *F*^2^. The threshold expression of *F*^2^ > σ(*F*^2^) is used only for calculating *R*-factors(gt) *etc*. and is not relevant to the choice of reflections for refinement. *R*-factors based on *F*^2^ are statistically about twice as large as those based on *F*, and *R*- factors based on ALL data will be even larger.

### Fractional atomic coordinates and isotropic or equivalent isotropic displacement parameters (Å^2^)

**Table:**

	*x*	*y*	*z*	*U*_iso_*/*U*_eq_	
Cl1	0.94869 (7)	1.35122 (11)	0.60169 (13)	0.0725 (4)	
N1	0.57886 (16)	0.7625 (2)	0.3769 (3)	0.0405 (7)	
H1	0.5945	0.6791	0.4057	0.049*	
N2	0.63579 (17)	0.8715 (2)	0.4127 (3)	0.0375 (6)	
N3	0.47946 (16)	0.9177 (2)	0.2451 (3)	0.0429 (7)	
H3	0.5175	0.9828	0.2719	0.051*	
N4	0.39947 (16)	0.9500 (2)	0.1494 (3)	0.0434 (7)	
H4A	0.4001	0.9058	0.0443	0.065*	
H4B	0.3513	0.9224	0.2129	0.065*	
O1	0.44329 (14)	0.6900 (2)	0.2677 (3)	0.0460 (6)	
C1	0.4970 (2)	0.7867 (3)	0.2949 (4)	0.0358 (7)	
C2	0.7519 (2)	0.7025 (3)	0.5085 (4)	0.0518 (9)	
H2A	0.7062	0.6479	0.5682	0.078*	
H2B	0.8056	0.7075	0.5825	0.078*	
H2C	0.7669	0.6601	0.3955	0.078*	
C3	0.7158 (2)	0.8469 (3)	0.4762 (4)	0.0357 (7)	
C4	0.77306 (19)	0.9705 (3)	0.5105 (4)	0.0353 (7)	
C5	0.8596 (2)	0.9605 (3)	0.5861 (4)	0.0458 (8)	
H5	0.8821	0.8733	0.6173	0.055*	
C6	0.9131 (2)	1.0768 (4)	0.6161 (4)	0.0511 (9)	
H6	0.9710	1.0676	0.6663	0.061*	
C7	0.8802 (2)	1.2056 (3)	0.5714 (4)	0.0443 (8)	
C8	0.7950 (2)	1.2196 (4)	0.4976 (4)	0.0513 (9)	
H8	0.7729	1.3074	0.4678	0.062*	
C9	0.7423 (2)	1.1031 (3)	0.4676 (4)	0.0475 (9)	
H9	0.6845	1.1136	0.4173	0.057*	

### Atomic displacement parameters (Å^2^)

**Table:**

	*U*^11^	*U*^22^	*U*^33^	*U*^12^	*U*^13^	*U*^23^
Cl1	0.0749 (7)	0.0666 (7)	0.0758 (7)	−0.0359 (5)	−0.0187 (5)	0.0017 (5)
N1	0.0364 (16)	0.0234 (14)	0.0618 (18)	0.0002 (12)	−0.0087 (13)	0.0031 (12)
N2	0.0367 (15)	0.0287 (15)	0.0471 (15)	−0.0058 (12)	−0.0022 (12)	−0.0013 (11)
N3	0.0377 (16)	0.0238 (15)	0.0671 (18)	−0.0020 (12)	−0.0125 (13)	0.0000 (12)
N4	0.0361 (15)	0.0335 (15)	0.0605 (17)	0.0027 (12)	−0.0060 (12)	0.0004 (12)
O1	0.0397 (13)	0.0231 (12)	0.0753 (16)	−0.0055 (10)	−0.0085 (11)	0.0006 (10)
C1	0.0372 (19)	0.0216 (18)	0.0488 (19)	0.0004 (14)	0.0016 (15)	−0.0018 (14)
C2	0.055 (2)	0.043 (2)	0.058 (2)	0.0051 (18)	−0.0117 (17)	−0.0020 (16)
C3	0.0360 (19)	0.0370 (18)	0.0342 (16)	0.0020 (15)	−0.0013 (14)	−0.0022 (14)
C4	0.0332 (18)	0.0379 (19)	0.0349 (17)	−0.0019 (14)	−0.0016 (14)	−0.0011 (14)
C5	0.042 (2)	0.044 (2)	0.051 (2)	−0.0002 (17)	−0.0057 (16)	0.0064 (16)
C6	0.041 (2)	0.061 (3)	0.052 (2)	−0.0070 (19)	−0.0125 (16)	0.0029 (18)
C7	0.045 (2)	0.045 (2)	0.0431 (19)	−0.0125 (17)	−0.0041 (16)	−0.0033 (15)
C8	0.052 (2)	0.037 (2)	0.065 (2)	−0.0075 (17)	−0.0142 (18)	0.0026 (16)
C9	0.0366 (19)	0.042 (2)	0.064 (2)	−0.0034 (16)	−0.0142 (16)	0.0021 (17)

### Geometric parameters (Å, °)

**Table:**

Cl1—C7	1.735 (3)	C2—H2B	0.9600
N1—C1	1.364 (4)	C2—H2C	0.9600
N1—N2	1.364 (3)	C3—C4	1.476 (4)
N1—H1	0.8600	C4—C5	1.388 (4)
N2—C3	1.285 (4)	C4—C9	1.387 (4)
N3—C1	1.336 (3)	C5—C6	1.382 (4)
N3—N4	1.404 (3)	C5—H5	0.9300
N3—H3	0.8600	C6—C7	1.368 (4)
N4—H4A	0.8900	C6—H6	0.9300
N4—H4B	0.8900	C7—C8	1.369 (4)
O1—C1	1.234 (3)	C8—C9	1.378 (4)
C2—C3	1.502 (4)	C8—H8	0.9300
C2—H2A	0.9600	C9—H9	0.9300
			
C1—N1—N2	119.5 (2)	N2—C3—C2	123.3 (3)
C1—N1—H1	120.2	C4—C3—C2	121.1 (3)
N2—N1—H1	120.3	C5—C4—C9	116.9 (3)
C3—N2—N1	119.1 (2)	C5—C4—C3	122.1 (3)
C1—N3—N4	120.5 (2)	C9—C4—C3	121.0 (3)
C1—N3—H3	119.7	C6—C5—C4	121.7 (3)
N4—N3—H3	119.7	C6—C5—H5	119.1
N3—N4—H4A	109.2	C4—C5—H5	119.1
N3—N4—H4B	109.1	C7—C6—C5	119.5 (3)
H4A—N4—H4B	109.5	C7—C6—H6	120.2
O1—C1—N3	122.8 (3)	C5—C6—H6	120.2
O1—C1—N1	120.3 (3)	C6—C7—C8	120.4 (3)
N3—C1—N1	116.9 (3)	C6—C7—Cl1	119.6 (2)
C3—C2—H2A	109.5	C8—C7—Cl1	119.9 (3)
C3—C2—H2B	109.5	C7—C8—C9	119.7 (3)
H2A—C2—H2B	109.5	C7—C8—H8	120.2
C3—C2—H2C	109.5	C9—C8—H8	120.2
H2A—C2—H2C	109.5	C8—C9—C4	121.8 (3)
H2B—C2—H2C	109.5	C8—C9—H9	119.1
N2—C3—C4	115.6 (3)	C4—C9—H9	119.1

### Hydrogen-bond geometry (Å, °)

**Table:**

*D*—H···*A*	*D*—H	H···*A*	*D*···*A*	*D*—H···*A*
N1—H1···N4^i^	0.86	2.24	3.024 (3)	152
N3—H3···O1^ii^	0.86	2.09	2.850 (3)	147
N4—H4A···O1^iii^	0.89	2.34	3.206 (3)	164

Symmetry codes: (i) −*x*+1, *y*−1/2, −*z*+1/2; (ii) −*x*+1, *y*+1/2, −*z*+1/2; (iii) *x*, −*y*+3/2, *z*−1/2.
